# Lysophosphatidic Acid Induced Apoptosis, DNA Damage, and Oxidative Stress in Spinal Cord Neurons by Upregulating LPA4/LPA6 Receptors

**DOI:** 10.1155/2022/1818758

**Published:** 2022-09-30

**Authors:** Yifan Yang, Jing Xu, Qingxin Su, Yiran Wu, Qizheng Li, Zongren Ma, Tao Ding

**Affiliations:** ^1^Department of Rehabilitation Medicine, First Affiliated Hospital of Kunming Medical University, No 295 Xichang Road, Xishan District, Kunming 650032, China; ^2^Department of Traditional Chinese Medicine, First Affiliated Hospital of Kunming Medical University, No 295 Xichang Road, Xishan District, Kunming 650032, China

## Abstract

Lysophosphatidic acid (LPA) has disruptive effects on lumbar spinal stenosis (LSS). Recently, LPA has been reported to be involved in spinal cord neuronal injury and toxicity, promoting the pathogenesis of LSS. However, the exact effects of LPA on spinal cord neurons remain unknown. The purpose of this study is to investigate the effects of LPA (18 : 1) on spinal cord neuronal cytotoxicity, apoptosis, DNA damage, and oxidative stress. After clinical detection of LPA secretion, spinal cord neurons were treated with LPA (18 : 1); cell viability was analyzed by MTT assay, and LDH leakage was detected by LDH kit; cell apoptosis was detected by flow cytometry; ROS production was measured by DCFDA staining and MitoSOX Red Staining; the activation of the G*α*12/G*α*13 signaling pathway was detected by serum response factor response element (SRF-RE) luciferase reporter gene; the relationship among LPA, LPA4/6, and ROCK was examined by western blotting. In spinal cord neurons treated with LPA (18 : 1), cellular activity decreased and LDH release increased. The Rho kinase inhibitor (Y-27632) can attenuate LPA-induced apoptosis, DNA damage, and oxidative stress in spinal cord neurons. Moreover mechanistic investigation indicated that LPA (18 : 1) activates G*α*12/13–Rho–ROCK2-induced apoptosis, DNA damage, and oxidative stress in spinal cord neurons by upregulating LPA4/LPA6 receptors. Further, the Rho kinase inhibitor Y-27632 attenuates the effects of LPA by downregulating LPA4/LPA6 receptors. Taken together, the possible mechanism by which LPA secretion in LSS patients aggravates patient injury was further elucidated using an LPA-induced spinal cord neuronal injury cell model in vitro.

## 1. Introduction

Lumbar spinal stenosis (LSS) is a common spinal degenerative clinical disease and one of the clinical manifestations of neuropathic pain [1]. Most symptoms of LSS are attributed to compression of the spinal cord, nerve roots, or cauda equina [2]. Compression of cauda equina fibers in patients with LSS causes hypersensitivity and sensitization in the central nervous system (CNS) and peripheral nervous system (PNS), affecting millions of people worldwide with severe debilitating neuropathic pain [3]. Clinically, the most common symptom of LSS is neurogenic claudication, where pain and discomfort from nerve compression radiate from the spine to the legs, causing the patient to lose sensation, fatigue, and balance problems [4]. Although current clinical treatments for LSS yield favorable outcomes (surgical and nonsurgical), the underlying pathophysiological mechanisms of LSS remain poorly defined.

Lysophosphatidic acid (LPA), a lysophospholipid found in body fluids such as serum and cerebrospinal fluid (CSF) [5], is expressed at different levels in different tissues (such as the brain) to activate G protein-coupled receptors and regulate cellular survival, proliferation, differentiation, and other biological functions [6, 7]. Previous studies have demonstrated that LPA plays a key role in the development and maintenance of neuropathic pain. Clinically, LPA is significantly increased in the early stages of patients with neuropathic pain and is associated with clinical symptoms of patients [8]. In neural mechanisms, LPA produced by autotaxin (ATX) affects the central nervous system (CNS) by regulating neural progenitor physiology, neuronal cell death, axon retraction, and inflammation, further causing neurological damage and neuropathic pain [9]. Recent studies have found that the level of LPA in the CSF of LSS patients was significantly increased [1]. Meanwhile, another study found that LPA promoted apoptosis of spinal cord neurons and mediated LSS injury [10]. However, the molecular mechanisms of LPA involved in LSS remain obscure.

Rho kinase (ROCK) regulates various biological functions such as gene transcription, translation, cycle progression, neuronal survival, dendrite outgrowth, spine maturation, and axon guidance by binding to GTP [11, 12]. A recent study found that myelin-derived proteins inhibit neurite formation by activating Rho. At the same time, Rho kinase inhibitors can induce neurite and axon growth in the spinal cord and brain after nervous system injury and promote nerve injury repair [13, 14]. In addition, increasing evidence suggests that inhibition of the Rho pathway may be effective in the treatment of spinal cord nerve injury [11]. Although Rho kinase inhibitors are used clinically in the treatment of cerebrovascular diseases to promote blood flow improvement and neuroprotection, the specific mechanism of action of Rho kinase inhibitors in LSS is unclear.

In this study, we explored the effects of the Rho kinase inhibitor-Y-27632 on cytotoxicity, apoptosis, DNA damage, and oxidative stress in spinal cord neurons treated by LPA (18 : 1). In this study, we found that LPA activates G*α*12/13–Rho–ROCK2-induced apoptosis, DNA damage, and oxidative stress in spinal cord neurons by upregulating LPA4/LPA6 receptors. Furthermore, the Rho kinase inhibitor Y-27632 attenuated the effects of LPA by downregulating LPA4/LPA6 receptors.

## 2. Materials and Methods

### 2.1. Patient Characteristics

We included 23 LSS patients treated at the First Affiliated Hospital of Kunming Medical University between December 2016 and May 2019 (13 males and 10 females; mean age, 70.9 years; range, 49–81 years), and 13 idiopathic scoliosis patients, who had no neurological symptoms, were used as a control group (8 males and 5 females, mean age: 72 ± 4 years; range, 51–80 years). The study was approved by the Ethics Committee of the First Affiliated Hospital of Kunming Medical University (2016 Ethics Application L No. 48), obtained the informed consent from the patients, and was carried out in accordance with the Helsinki Declaration.

### 2.2. Samples

In patients with LSS identified by myelography, CSF was collected by lumbar puncture, and idiopathic scoliosis patients who had no neurological symptom were used as a control group. The collected CSF were stored at -80°C until measurement.

### 2.3. Measurement of LPA and LPC

1 mL of cerebrospinal fluid from patients were collected and stored at -80°C. Quantification of LPA and LPC was performed as described by Okudaira et al. [15, 16]. Briefly, after centrifugation of the CSF samples mixed with methanol, the supernatant was taken for LC-MS analysis. LC (Shiseido) separation was performed using a Capcell Pak ACR phase column (1.5 mm × 250 mm, 3 *μ*m particle size; Shiseido) with a gradient elution of solvent A (5 mM ammonium formate in water) and solvent B (5 mM ammonium formate in 95% (*v*/*v*) acetonitrile). For each lysophospholipid class, 3 kinds of LPA (16 : 0, 18 : 1, and 18 : 2) and 3 kinds of LPC (16 : 0, 18 : 1, and 18 : 2) were monitored.

### 2.4. ATX Activity Assay

To measure the level of ATX in CSF samples, we refer to the method of Omori et al. [5] and Umezu-Goto et al. [17], using LPC as the substrate to release the amount of choline to assess the level of ATX.

### 2.5. Materials

Lysophosphatidic acids (LPA18:1), 3-(4,5-dimethyl-2-thiazolyl)-2,5-diphenyltetrazolium bromide (MTT) and penicillin, and streptomycin were from Sigma-Aldrich (St. Louis, MO, USA). Rho kinase inhibitor Y-27632 was from Calbiochem (Darmstadt, Germany). Dulbecco's modified Eagle medium (DMEM) was purchased from Gibco (Grand Island, NY, USA). All antibodies were purchased from Signaling Technology (MA, USA) except ROCK1/2 which were purchased from Abcam (Cambridge, MA, USA).

### 2.6. Isolation and Primary Culture of Spinal Cord Neurons

After the E13 pregnant Sprague-Dawley (SD) rats were euthanized, the spinal cord tissues were isolated from rat embryos. After the spinal cord tissue was washed once with PBS, digestion solution (0.1% trypsin) was added. After digestion, tissue suspension was filtered through 100-mesh, 200-mesh, and 400-mesh sieves. The filtrate was centrifuged at 300 g for 5 min to obtain cells. The cells were then resuspended in complete medium and seeded in polylysine-coated dishes. After 24 h of culture, the medium was changed to neural basal growth medium containing 2% B27, 1% N_2_, 2 mM glutamine, and 1 *μ*M cytarabine. Spinal cord neurons were divided into the following groups: the control group (normal culture), LPA group (treated with LPA for 2 h), Y-27632 group (treated with Y-27632 for 1 h), LPA+Y-27632 group (treated with LPA and Y-27632), si-LPA4/LPA6 group (transfected with the lentivirus of silencing LPA4/LPA6), and LPA+si-LPA4/LPA6 group (treated with LPA and transfected with corresponding silencing LPA4/LPA6).

### 2.7. MTT Assay

Spinal cord neurons (4000 cells/well) were plated in 96-well plates for 24 h. Neurons were treated or transfected with LPA, Y-27632, and si-LPA4/LPA6. Next, we added 20 *μ*L of MTT (5 mg/mL in phosphate-buffered saline) for another 4 h of incubation, then 100 *μ*L DMSO was applied to wells for 10 min. The OD value was obtained in a microplate reader (Thermo-Fisher Scientific, USA) at 570 nm.

### 2.8. Lactate Dehydrogenase (LDH) Release Assay

LDH activity was detected by LDH assay kit (Sigma-Aldrich) [18]. Neurons were cultured for 24 h after different treatments and transfections, and the supernatant from each well was collected. Then, the supernatant was incubated with 2,4-dinitrophenylhydrazine. The OD at 490 nm was measured by microplate reader (Thermo-Fisher Scientific, USA). Each experiment was repeated three times.

### 2.9. Flow Cytometry

Spinal cord neurons apoptosis was detected by Annexin V-FITC Kit. Neurons were cultured for 24 h after different treatments and transfections, trypsinized. After three washes with PBS, Incubate with Annexin V-FITC (5 *μ*L) and propidium iodide (10 *μ*L09) for 20 min. Finally, these apoptotic cells were analyzed using flow cytometry.

### 2.10. DCFDA Staining Assay

Neurons were treated or transfected with LPA, Y-27632, and si-LPA4/LPA6. Then, DCFDA assays were performed using a DCFDA Cellular ROS Assay Kit (Sigma-Aldrich, St. Louis, MO). According to the manufacturer's instructions, we incubate indifferently treated and transfected neurons with 10 *μ*M DCFDA dye for 20 min and trypsinize; the ROS generation was examined by the flow cytometer (Bender Med Systems, CA, USA).

### 2.11. LPA mRNA Assay

The LPA mRNA assay was carried out as previously described [19], and total RNA from the spinal cord neurons was isolated using TRIzol reagent (Invitrogen, Waltham, MA, USA). After detecting the RNA concentration, the extracted RNA was reverse transcribed to cDNA using a cDNA reverse transcription kit (Thermo Fisher Scientific, Waltham, MA, USA). The relative mRNA expression levels of LPA1, LPA2, LPA3, LPA4, LPA5, and LPA6 were detected by qPCR using SYBR Green qPCR Master Mix according to the manufacturer's instructions (Thermo Fisher Scientific, USA). Beta-actin were used as internal reference. Thermal cycling conditions were as follows: predenaturation 1 cycle at 95°C for 20 s and 40 cycles at 95°C for 20 s, 60°C for 30 s, and 72°C for 25 s. The LPA primer sequences are shown in Table 1.

### 2.12. Western Blotting

Proteins were isolated from differently treated and transfected spinal cord neurons by using RIPA lysis buffer (Invitrogen; USA). Then, the protein concentration was detected by bicinchoninic acid assay (Thermo Fisher Scientific, USA). Equivalent amounts of protein were separated by SDS-PAGE and transferred to polyvinylidene fluoride (PVDF) membranes. Next, the membrane was allowed to incubate with the following antibodies: LPA4 (1 : 1500), LPA6 (1 : 2000), ROCK1(1 : 1500), ROCK2 (1 : 1500), *γ*-H2AX (1 : 2000), and H2AX (1 : 2000). The next day, the membranes were washed with PBS and incubated with HRP-conjugated secondary antibody for 1 hour at room temperature. The target proteins were visualized using enhanced chemiluminescence kit (Thermo Fisher Scientific, USA), and the band intensities were obtained by densitometric analysis of images using ImageJ software.

### 2.13. SRF-RE Luciferase Assay

The SRF-RE luciferase assay was carried out as previously described [20]. In brief, neurons were cultured for 24 h after different treatments and transfections. Neurons were then transfected with 2 *μ*g SRF-RE luciferase-pGL4.35 (Promega) and 1 *μ*g SV40-Renilla luciferase-pRL (Promega) by transfection reagent (Clontech) and incubated. After 6 h of incubation, cells were cultured in DMEM containing 0.1% BSA for 12 h. Relative luciferase activity was measured using Dual Luciferase Reporter Assay System kit (Promega).

### 2.14. Statistical Analysis

All data were presented as mean values ± standard deviation (SD), and the results of the experiment are repeated three times. Statistical variances among multigroups were calculated through the one-way analysis of variance (ANOVA). Statistical analyses were performed by GraphPad Prism 7.0 software (GraphPad Software, Inc.). *p* < 0.05 or *p* < 0.01 was considered to indicate a statistically significant difference.

## 3. Results

### 3.1. High Expression of LPA in CFS of LSS Patients

**Figure 1:**
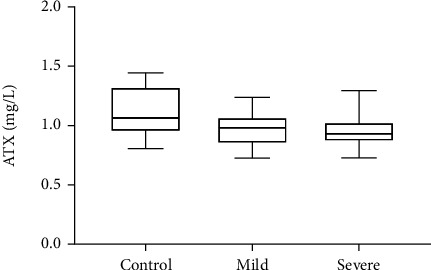
High expression of LPA and LPC in CFS of LSS patients. ATX levels in CSF between the severe group, mild group, and control group in the LSS.

We divided all patients (including control) into 3 categories based on the NPSI score [21]: the control group (the NPSI score was 0; *n* = 13), mild group (NPSI score ≤ 21; *n* = 11), severe group (NPSI score > 21; *n* = 12) [15]. LPA, a potent bioactive lipid mediator, is mainly produced from lysophosphatidylcholine (LPC) via autotaxin (ATX) [1]. Therefore, we examined the expression of LPA, LPC, and ATX in CSF of different patients. The test results showed that LSS patients had significantly higher levels of LPA and LPC and there was no significant difference in ATX levels, compared to controls. Among them, 3 kinds of LPA (16 : 0, 18 : 1, and 18 : 2) and 3 kinds of LPC (16 : 0, 18 : 1, and 18 : 2) were detected in the CSF of all subjects (Table 2, Table 3 and Figure 1). Based on these preliminary clinical results, we found that the 18 : 1 difference was the most significant, so we chose to focus on LPA (18 : 1).

### 3.2. LPA Effects on Cell Viability and Cytotoxicity in Spinal Cord Neurons

**Figure 2:**
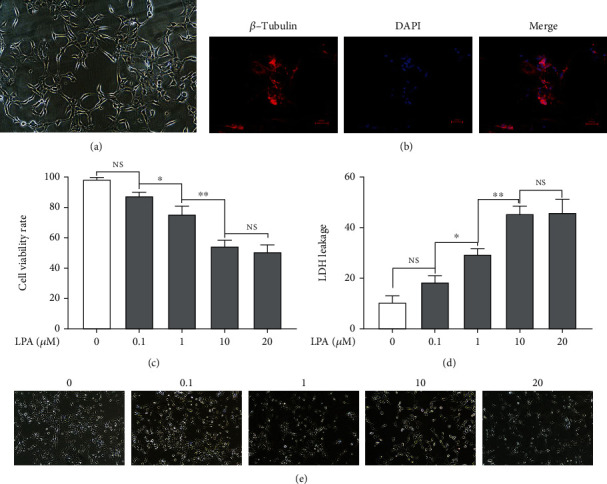
LPA effects on cell viability and cytotoxicity in spinal cord neurons. (a) Observation of primary spinal cord neurons with microscope (100x). (b) The expression of *β*-tubulin in spinal cord neurons were measured by Immunofluorescence (200x). (c, e) spinal cord neurons were treated with different concentrations of LPA for 24 h, the cell viability was measured by the 3-(4,5-dimethylthiazol-2-yl)-2,5-diphenyl tetrazolium bromide (MTT) assay, the LDH release was measured by LDH assay kit. (d) Cell viability was observed by microscope (100x). Asterisks indicate statistical significance (^∗^*p* < 0.05, ^∗∗^*p* < 0.01).

Under an optical microscope, the cells had a long and spindle-shaped morphology, with long neurites on both sides. In addition, *β*-tubulin as an identification indicator of spinal cord neurons, *β*-tubulin immunofluorescence showed that neuronal cell cytoplasm was rich in red, demonstrating a high expression of *β*-tubulin (Figures 2(a) and 2(b)).

In order to investigate the viability and cytotoxicity of LPA on spinal cord neurons, LDH and MTT tests were used to analyze the LDH release and viability of cells treated with 0, 0.1, 1, 10, and 20 *μ*M LPA for 24 h [10]. The results showed that LPA dose dependently increased LDH release and reduced cell viability compared with the untreated cell. However, under the action of LPA concentration of 20 *μ*M, the LDH release and cell viability of spinal cord neurons are not statistically significant from those of 10 *μ*M (Figures 2(c)–2(e)). According to these trial results, the LPA concentrations of 10 *μ*M were selected for further studies.

### 3.3. Rho Kinase Inhibitor Alleviated Cytotoxicity in the LPA-Induced Spinal Cord Neurons

**Figure 3:**
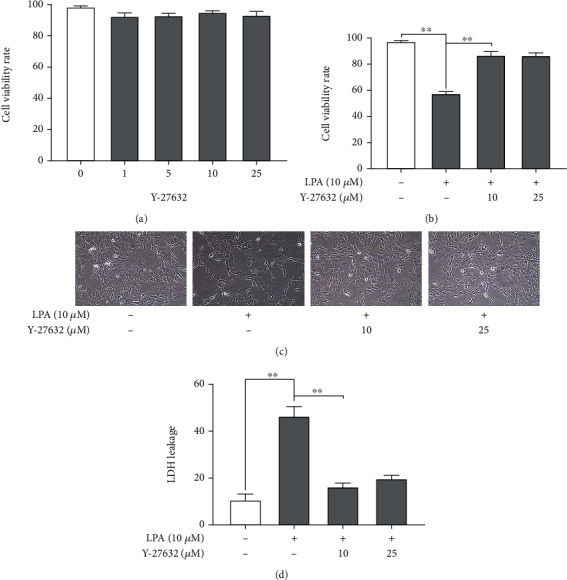
Rho kinase inhibitor alleviated cytotoxicity in the LPA-induced spinal cord neurons. (a, b) The cell viability was measured by MTT assay. (c) Cell viability was observed by microscope (100x). (d) The LDH release was measured by LDH assay kit. Asterisks indicate statistical significance (^∗^*p* < 0.05, ^∗∗^*p* < 0.01).

Previous studies have reported that Rho kinase inhibitors can alleviate nerve damage and promote neuronal regeneration, which is of great significance in the treatment of LSS and neurological diseases [22]. Rho kinase is one of the downstream signaling molecules of LPA-receptor. To determine whether the effect of LPA on spinal cord neurons is mediated by Rho kinase, we measured the response in the presence of the Rho kinase inhibitor Y-27632 (1, 5 10, and 25 *μ*M). The viability of spinal cord neurons were detected using the MTT assay. The treatment of Y-27632 (1, 5 10, and 25 *μ*M) had no significant effect on spinal cord neurons (Figure 3(a)). Additionally, MTT and LDH assay results showed that treatment with 10 *μ*M and 25 *μ*M Y-27632 significantly attenuated LPA (10 *μ*M)-induced decrease in cell viability and increase in LDH release (Figures 3(b)–3(d)). These results indicate that Rho kinase is related to spinal cord neuron cytotoxicity and viability induced by LPA.

### 3.4. Rho Kinase Inhibitor Alleviated Apoptosis, DNA Damage, and Oxidative Stress in the LPA-Induced Spinal Cord Neurons

**Figure 4:**
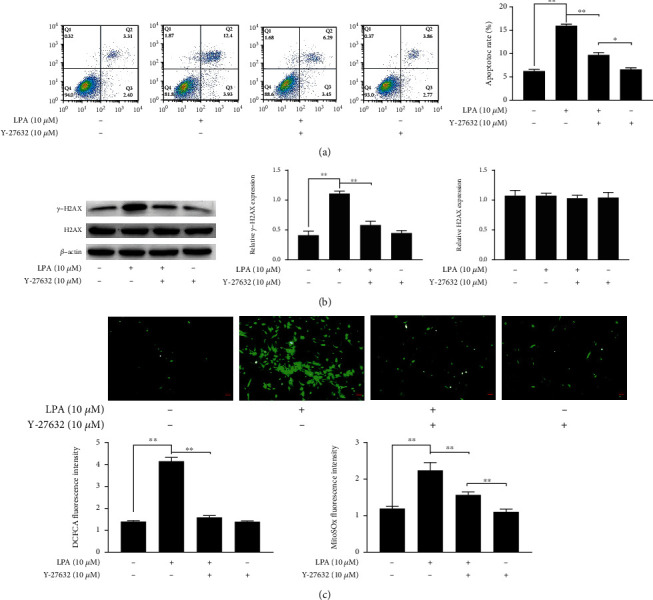
Rho kinase inhibitor alleviated apoptosis, DNA damage, and oxidative stress in the LPA-induced spinal cord neurons. (a) The apoptotic ratio was detected by flow cytometry. (b) The expression of DNA damage-related proteins in spinal cord neurons were measured by western blotting. (c) Quantity analysis of intensity of the DCFDA staining and MitoSOx staining. Asterisks indicate statistical significance (^∗^*p* < 0.05, ^∗∗^*p* < 0.01).

LAP-induced cytotoxicity, is attributed to oxidative stress; in addition, oxidative stress also induces DNA damage and apoptosis [23]. Therefore, we measured the number of apoptotic cells of spinal cord neurons treated with LPA and Y-27632 (10 *μ*M). Rho-associated kinase inhibitor (Y-27632) attenuated LPA-induced apoptosis of spinal cord neurons when compared with the control group (Figure 4(a)). Simultaneously, activation of oxidative stress and DNA damage play critical roles in spinal cord neuron apoptosis [24]. Therefore, we measured the effects of LPA and Y-27632 on oxidative stress and DNA damage. Western blotting assays demonstrated that Y-27632 downregulated LAP-induced *γ*-H2AX expression level but did not affect H2AX expression. Additionally, LPA increased the reactive oxygen species (ROS) production and increased fluorescence intensity of DCFDA and MitoSOX in spinal cord neurons; however, Y-27632 could decrease ROS induced by LPA (Figures 4(b)–4(d)). These results suggest that Y-27632 decreases ROS, apoptosis rate, and *γ*-H2AX expression level; finally, it prevents apoptosis, DNA damage, and oxidative stress induced by LPA in spinal cord neurons.

### 3.5. LPA and Rho Kinase Inhibitors Affect the Expression of LPA4 and LPA6 in Spinal Cord Neurons

**Figure 5:**
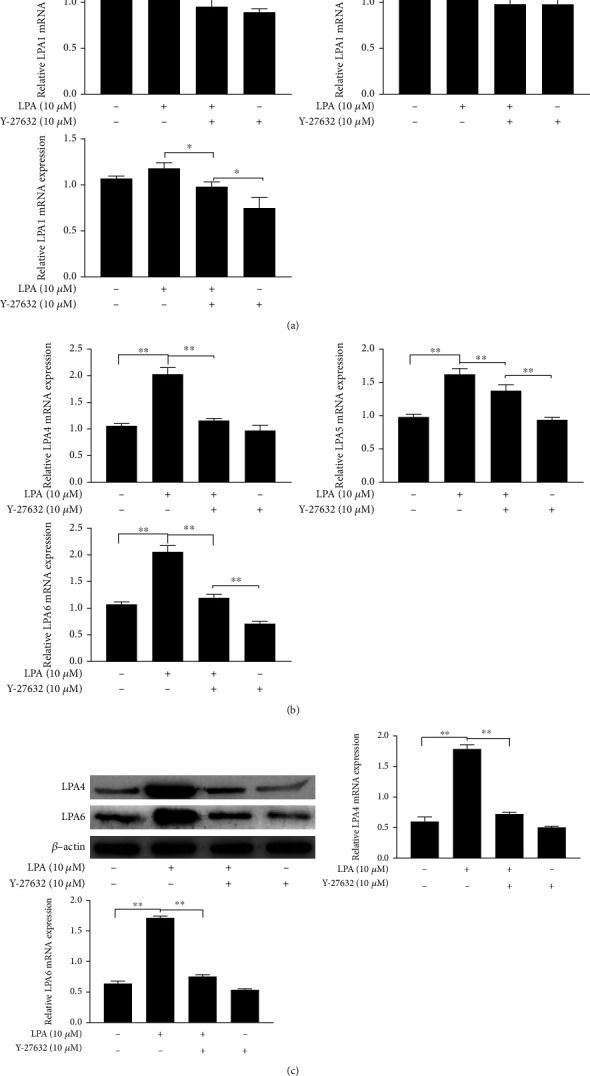
LPA and Rho kinase inhibitors affect the expression of LPA4 and LPA6 in spinal cord neurons. (a, b) The expression of LPA1, LPA2, LPA3, LPA4, LPA5, and LPA6 mRNAs in spinal cord neurons were measured by RT-qPCR. (c) The expression of LPA4 and LPA6 in spinal cord neurons were measured by western blotting. Asterisks indicate statistical significance (^∗^*p* < 0.05, ^∗∗^*p* < 0.01).

To identify the LPA receptors involved in spinal cord neurons injury, we tested LPA1, LPA2, LPA3, LPA4, LPA5, and LPA6 mRNA expressions. Among the six receptors of LPA, LPA1, LPA3, LPA4, LPA5, and LPA6 mRNA are upregulated by LPA (Figures 4(a) and 4(b)). When Y-27632 is used, it can significantly inhibit the expression of LPA4 and LPA6 mRNA (Figures 5(a) and 5(b)). This suggests that the alleviation of LPA-induced spinal cord neuron damaged by Rho kinase inhibitor may be related to the regulation of LPA4 and LPA6 expressions. Protein levels of LPA4 and LPA6 were further examined by western blotting indicating that the protein levels of LPA4 and LPA6 were downregulated after Y-27632 treatment, compared with the LPA-induced group (Figure 5(c)).

### 3.6. LPA4/LPA6 Mediates LPA-Induced Spinal Cord Neuron Apoptosis, DNA Damage, and Oxidative Stress

**Figure 6:**
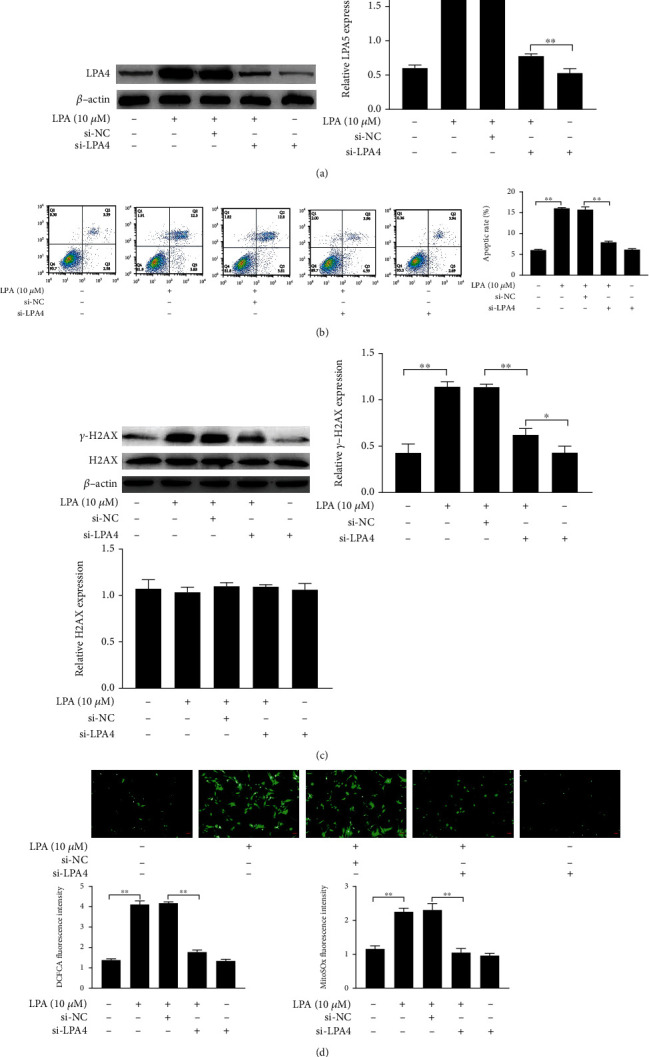
LPA4 mediates LPA-induced spinal cord neuron apoptosis, DNA damage, and oxidative stress. (a, c) The expression of LPA4, DNA damage-related proteins in spinal cord neurons were measured by western blotting. (b) The apoptotic ratio was detected by flow cytometry. (d) Quantity analysis of intensity of the DCFDA staining and MitoSOx staining. Asterisks indicate statistical significance (^∗^*p* < 0.05, ^∗∗^*p* < 0.01).

**Figure 7:**
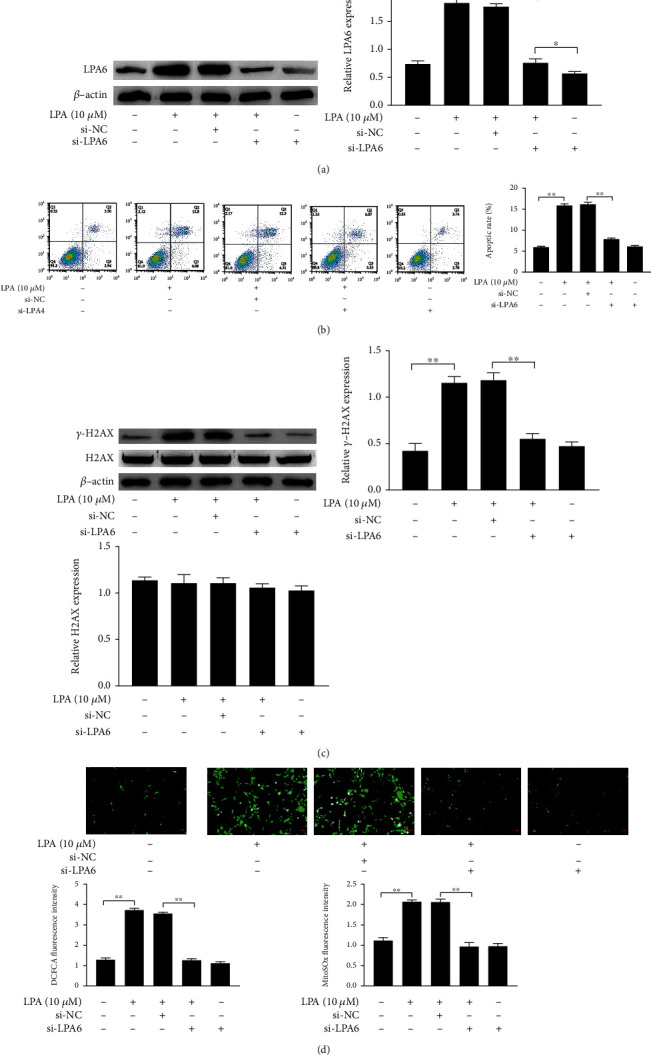
LPA6 mediates LPA-induced spinal cord neuron apoptosis, DNA damage, and oxidative stress. (a, c) The expression of LPA6 and DNA damage-related proteins in spinal cord neurons were measured by western blotting. (b) The apoptotic ratio was detected by flow cytometry. (d) Quantity analysis of intensity of the DCFDA staining and MitoSOx staining. Asterisks indicate statistical significance (^∗^*p* < 0.05, ^∗∗^*p* < 0.01).

To verify that LPA-induced apoptosis of spinal cord neurons, DNA damage, and oxidative stress are associated with LPA4/LPA6 receptors, we transfected si-NC, si-LPA4, and si-LPA6 into LPA-induced spinal cord neurons (12.5 nM each, 48 h pretreatment). The LPA4 and LPA6 were knockdown by si-LPA4 and si-LPA6 in spinal cord neurons (Figures 6(a) and 7(a)). We then further detected the effects of LPA4/LPA6 receptors on LPA-induced apoptosis, DNA damage, and oxidative stress in spinal cord neurons. si-LPA4 and si-LPA6 attenuated LPA-induced apoptosis of spinal cord neurons when compared with the LPA-Induced group (Figures 6(b) and 7(b)). Western blotting assays demonstrated that si-LPA4 and si-LPA6 downregulated LAP-induced *γ*-H2AX expression level. Additionally, si-LPA4 and si-LPA6 decreased fluorescence intensity of DCFDA and MitoSOX in LAP-induced spinal cord neurons (Figures 6(c), 6(d), 7(c), and 7(d)). The results of si-LPA4 and si-LPA6 transfection were consistent with those of Y-27632 treatment, which indicated that LPA-induced spinal cord neuron apoptosis, DNA damage, and oxidative stress were achieved through upregulation of the receptors LPA4 and LPA6.

### 3.7. LPA Activates G*α*12/13–Rho–ROCK2 by Upregulating LPA4 and LPA6

**Figure 8:**
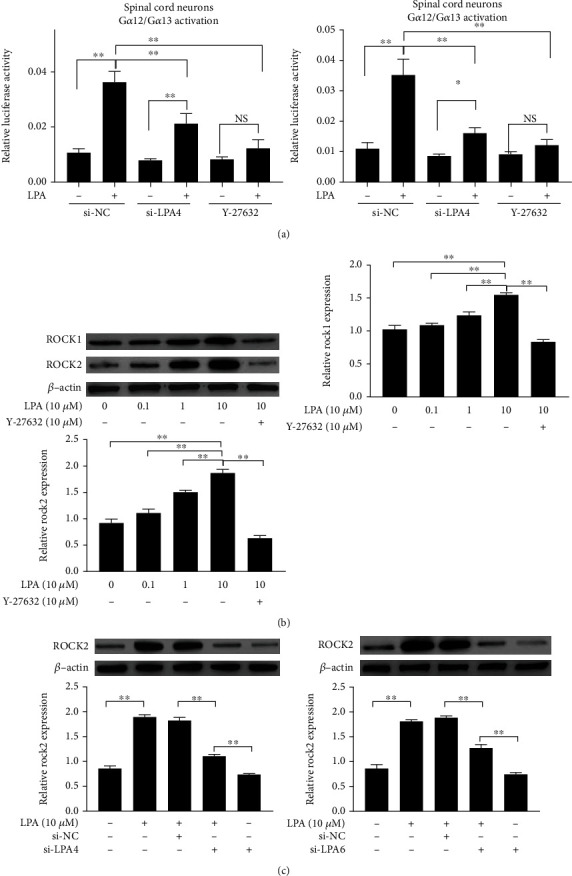
LPA activates G*α*12/13–Rho–ROCK2 by upregulating LPA4 and LPA6. (a) Activation of the G*α*12/13 pathway is detected by the serum response factor response element (SRF-RE) luciferase reporter gene. (b, c) The expression of ROCK1 and ROCK2 in spinal cord neurons were measured by western blotting. Asterisks indicate statistical significance (^∗^*p* < 0.05, ^∗∗^*p* < 0.01).

In LPA-induced spinal cord neurons, LPA4 and LPA6 receptors were upregulated. LPA4/LPA6, as G*α*12/13-coupled LPA receptors, could regulate the G*α*12/13 pathway [20, 25]. Therefore, we further detected the activation of the G*α*12/13 pathway in spinal cord neurons of each group. Next, we performed a serum response factor–responsive element (SRF-RE) luciferase reporter assay, which detects the activation of the G*α*12/G*α*13 signaling pathway [26]. LPA increased the reporter activity, which was abolished by the Rho inhibitor Y-27632 and the siRNA-mediated knockdown of LPA4 and LPA6 (Figure 8(a)). LPA-induced upregulation of ROCK2 expression; ROCK2 changed more significantly than ROCK1; furthermore, LPA-induced upregulation of ROCK2 was abolished by Rho inhibitor Y-27632 and siRNA-mediated knockdown of LPA4 and LPA6 (Figures 8(b) and 8(c)). These results suggest that LPA activates the G*α*12/13-Rho-ROCK2 pathway, by upregulating LPA4 and LPA6.

## 4. Discussion

The secretion of LPA in CSF is increased in LSS patients, but the type of LPA that plays a role in LSS [15], the receptor and the regulatory mechanism of LPA remain unclear. In this study, we identified LPA (18 : 1) as the predominant secretory type and LPA4 and LPA6 as LPA receptors responsible for spinal cord neuronal injury through clinical research. The results showed the consequences of the LPA pathway, linking it directly to apoptosis, DNA damage, and oxidative stress. Also, it was noticed that the Rho kinase inhibitor Y-27632 could downregulate the expression of LPA4 and LPA6 in spinal cord neurons and reduce LPA-induced spinal cord neuron injury. Finally, LPA-induced neuronal apoptosis, DNA damage, and oxidative stress in spinal cord neurons are caused by LPA upregulating the expression of LPA4 and LPA6 which further activates G*α*12/13-Rho-ROCK2. This study is the first to reveal one of the mechanisms by which LPA induces spinal neuronal injury, which may have therapeutic implications for LSS patients.

Compression of cauda equina fibers in patients with LSS causes hypersensitivity of the CNS and PNS leading to neuropathic pain [27]. The environment and mechanism of LSS inducing neuropathic pain occurrence are complex. Previous findings from LSS clinical patients and mouse models of neuropathic pain suggest that LPA signaling is the decisive mechanism by which LSS induces neuropathic pain [1, 28]. LPA, a potent bioactive lipid mediator, is mainly produced from LPC via ATX. In clinical studies, LPC and LPA are increased in the cerebrospinal fluid, plasma, and spinal cord tissue samples in LSS patients [29–31]. Similarly, in this study, LPA and LPC levels were significantly elevated in the CSF of LSS patients, while ATX levels were not significantly different. LPA species was converted from the corresponding LPC species with the action of ATX. This also suggests that changes in LPC levels might affect LPA levels to a greater extent than ATX levels. Among LPA species subtypes critical for neuropathic pain, 16 : 0, 16 : 1, 18 : 0, 18 : 1, 18 : 2, 20 : 4, and 22 : 6 were strongly associated with neuropathic pain and claudication severity in LSS [15, 32, 33]. In this study, we identified LPA (16 : 0, 18 : 1, and 18 : 2) as the predominant secretory type, where the 18 : 1 difference is more pronounced. This result is consistent with the findings of Lin et al., which may be attributed to the higher affinity of the enzyme ATX, which catalyzes the conversion of LPC to LPA, for 18 : 1 LPC [32]. LPA (18 : 1) is the predominant molecular specie of spinal LPA production after nerve injury; it has the strongest correlation with neuropathic pain and has been shown to be a key in animal models of neuropathic pain [32]. Thus, we will use LPA (18 : 1) for subsequent cell experiments.

LSS pathology is associated with inflammation, oxidative damage, and neuron death [34]. During the development of LSS, the blood-spinal cord barrier (BSCB) is disrupted, and the released inflammatory factors cause neuronal oxidative stress and apoptosis leading to loss of neurological functional [35]. In addition, DNA damage-dependent cell death is also a major cause of neurological diseases [36]. We isolated rat spinal cord neurons and treated them with LPA (18 : 1) and found that LPA (18 : 1) promoted spinal cord neuronal apoptosis, DNA damage, and oxidative stress. But the specific mechanism needs to be further explored.

We measured mRNA expression levels of 6 LPA receptor in LPA (18 : 1)-acted spinal neurons and revealed differences in mRNA expression patterns of LPA receptor subtypes. Our results showed that the expression levels of LPA4 mRNA and LPA6 mRNA were higher in LPA (18 : 1)-acted spinal neurons. In previous studies of LPA-acting receptors, LPAR1/3 receptors mediate some neuropathic pain mediated by LPA. Drug-induced neuropathic pain is alleviated in LPAR1 and LPAR3 knockout mice and in mice pretreated with the LPAR1/3 antagonist Ki16425 [37, 38]. However, in this study, we found that LPAR1/3 changes were not as pronounced as LPAR4/6 after LPA treatment of spinal cord neurons. LPA4/6 are all expressed in the brain as receptors of LPA, but previous studies only found the regulation of LPA1/3/5 on neuropathic pain but did not find the role of LPA4/6 in the development of LSS and neuropathic pain. Our results confirmed that LPA4/6, as the main receptors of LPA (18 : 1), played a major role in LPA (18 : 1)-induced apoptosis and cytotoxicity of spinal cord neurons. LPA promotes spinal cord neuron apoptosis, DNA damage, and oxidative stress. During this process, LPAR4/6 is highly expressed. Inhibited LPAR4/6 in spinal cord neurons can alleviate the effect of LPA (18 : 1) on spinal cord neuron apoptosis, DNA damage, and oxidative stress promotion.

Rho kinase is involved in neuropathic diseases of the central nervous system, including neuropathic pain [39, 40]. Previous studies have reported the role of Rho kinases on neuropathic pain through the Rho/ROCK cascade [41, 42]. One of these studies also demonstrated that a Rho kinase inhibitor improved motor dysfunction and pain perception in rats with lumbar spinal stenosis [13]. In addition, LPA acts as a physiological activator of Rho to activate ROCK through the LPAR receptor [43], ROCK is a ubiquitously expressed kinase with two known isoforms, ROCK1 and ROCK2 [44]. ROCK2 mRNA is widely distributed all along with the central nervous system [45], Interfering with ROCK2 mRNA and downregulating ROCK2 expression can improve neurological diseases including neuropathic pain [42]. This result is partially consistent with our current finding that LPA (18 : 1)-induced ROCK2 upregulation with less effect on ROCK1. In addition, we further demonstrated that Rho is activated under the action of LPA4/6, and interfering with LPA4/6 expression or using the Rho kinase inhibitor Y-27632 can alleviate the effects of LPA (18 : 1) on spinal cord neuronal apoptosis, DNA damage, and promotion of oxidative stress role.

In summary, the present study showed that LPA (18 : 1) activates G*α*12/13–Rho–ROCK2-induced apoptosis, DNA damage, and oxidative stress in spinal cord neurons by upregulating LPA4/LPA6 receptors. Further, the Rho kinase inhibitor Y-27632 attenuates the effects of LPA by downregulating LPA4/LPA6 receptors. In conclusion, the possible mechanism by which LPA secretion in LSS patients aggravates patient injury was further elucidated using an LPA-induced spinal cord neuronal injury cell model in vitro.

## Figures and Tables

**Table 1:** RNA primer sequences.

Gene	RNA primer sequences
LPA1 (forward primer)	GCCTTCCAGCATCCTAACA
LPA1 (reverse primer)	ACCCGACCGACCACACTCA
LPA2 (forward primer)	GATGGATTTCCGCAGGCGAACG
LPA2 (reverse primer)	CGCACCACCAACTGAAAGTGCC
LPA3 (forward primer)	CTATGTCGCCAACGTGATCC
LPA3 (reverse primer)	CTAGCGTACCGGTATACTTA
LPA4 (forward primer)	GCCTTCGAACAGTCCGCCCG
LPA4 (reverse primer)	GGAGATGTGCCGCGTCATGC
LPA5 (forward primer)	ATCCGCTTGCAAATACGCTC
LPA5 (reverse primer)	GACTTGCGGGTATAACGGCC
LPA6 (forward primer)	ATGGCCTCGGTTTTCTAACG
LPA6 (reverse primer)	ATGCGCGACATTGGCCTGCG
*β*-Actin (forward primer)	TCGCCATCCTGAATCTGGAGGCG
*β*-Actin (forward primer)	AGCTCTTAGGAGCAATCGCCCTT

**Table 2:** LPA and LPC levels in CSF between LSS patients and controls.

	Control (*n* = 13)	LSS (*n* = 23)	*p*
LPA			
16 : 0	0.0232	0.0411	<0.01
18 : 1	0.00678	0.0319	<0.01
18 : 2	0.000951	0.00449	<0.01
LPC			
16 : 0	0.000131	0.0000781	<0.01
18 : 1	0.00166	0.000486	<0.01
18 : 2	0.00179	0.000521	<0.01

LSS: lumbar spinal stenosis; LPA: lysophosphatidic acid; LPC: lysophosphatidylcholine.

**Table 3:** LPA and LPC levels in CSF between the severe and mild groups with NPSI in the LSS.

	NPSI		
LPA	Mild group (*n* = 11)	Severe group (*n* = 12)	*p*
16 : 0	0.0425	0.0435	0.0723
18 : 1	0.0228	0.0326	<0.01
18 : 2	0.00431	0.00441	0.0213
LPC			
16 : 0	0.000131	0.000132	0.02
18 : 1	0.00166	0.00167	0.0344
18 : 2	0.00178	0.00179	0.0218

LSS: lumbar spinal stenosis; LPA: lysophosphatidic acid; LPC: lysophosphatidylcholine.

## Data Availability

All datasets presented in this study are included in the article. All data are real and guarantee the validity of experimental results.
